# Overview of medical physics education and research programs in a non‐academic environment

**DOI:** 10.1002/acm2.14124

**Published:** 2023-08-21

**Authors:** Jessica M. Fagerstrom, Thomas A. D. Brown, Darryl G. L. Kaurin, Saikanth Mahendra, M. Miron Zaini

**Affiliations:** ^1^ Department of Radiation Oncology University of Washington Seattle Washington USA; ^2^ Department of Radiation Oncology Maine Medical Center Portland Maine USA; ^3^ Northwest Medical Physics Center Lynnwood Washington USA

**Keywords:** curriculum, education, medical physics, residency, teaching

## Abstract

Northwest Medical Physics Center (NMPC) is a nonprofit organization that provides clinical physics support to over 35 radiation therapy facilities concentrated in the Pacific Northwest. Although clinical service is the primary function of NMPC, the diverse array of clinical sites and physics expertise has allowed for the establishment of structured education and research programs, which are complementary to the organization's clinical mission. Three clinical training programs have been developed at NMPC: a therapy medical physics residency program accredited by the Commission on Accreditation of Medical Physics Education Programs (CAMPEP), an Applied Physics Technologist (APT) program, and a summer undergraduate internship program. A partnership has also been established with a major radiation oncology clinical vendor for the purposes of validating and testing new clinical devices across multiple facilities. These programs are managed by a dedicated education and research team at NMPC, made up of four qualified medical physicists (QMPs). The education and research work has made a significant contribution to the organization's clinical mission, and it has provided new training opportunities for early‐career physicists across many different clinical environments. Education and research can be incorporated into nonacademic clinical environments, improving the quality of patient care, and increasing the number and type of training opportunities available for medical physicists.

## 1 INTRODUCTION

In this manuscript, we describe the development of a formalized research program and three education programs in a clinical environment outside a university and teaching‐hospital setting. To the best of our knowledge, there is currently no published literature regarding the design and execution of these types of medical physics programs in an environment where the main emphasis is clinical service. There are unique administrative and resource management challenges in an environment that is not fundamentally designed for research and education. This paper is intended to provide a template for other interested medical physics service groups, using a theoretical framework based on guiding learners to build progressing levels of clinical skills, competence, and performance.

## 2 LEARNING OBJECTIVES

1. To understand the benefits of an education and research program in a medical physics group whose primary mission is clinical service.
2. To understand the design and execution of clinical training and research programs in a nonacademic environment.

## 3 THEORETICAL FRAMEWORK

**FIGURE 1:**
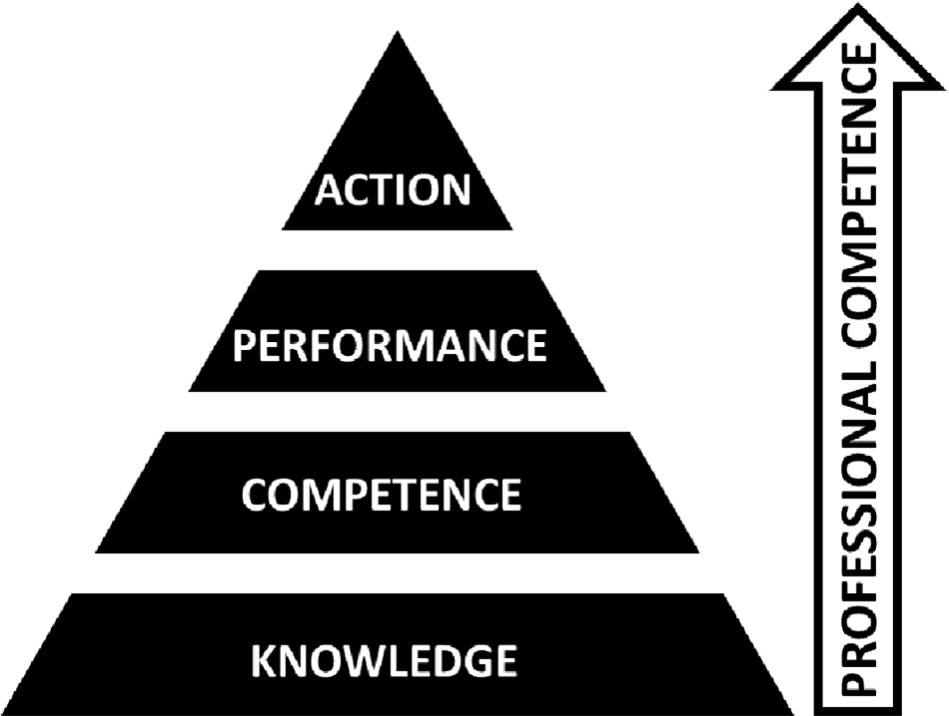
Image illustrating Miller's pyramid of clinical competence. Learners climb four hierarchical levels of proficiency: first, basic factual and conceptual knowledge; second, procedural knowledge, representing the ability to apply knowledge for problem solving; third, demonstration of skill, indicating the ability to perform tasks under direct supervision or in a testing environment; and finally, independent clinical practice in authentic contexts. Adapted from G.E. Miller, “The assessment of clinical skills/competence/performance,” *Academic Medicine*, 65(9), S63, 1990. Copyright 1990, Association of American Medical Colleges.

A theoretical framework is a foundation that helps situate scholarly work within a theoretical context or model.^1^ Bordage^2^ describes theoretical frameworks as arising from “theories with well‐organised principles and propositions that have been confirmed by observations or experiments; models derived from theories, observations or sets of concepts; or evidence‐based best practices derived from outcome and effectiveness studies.” The theoretical framework that best describes the perspective from which this work was approached is a model commonly used in medical education described by Miller^3^ for the assessment of clinical skills, competence, and performance. In this model, the learner progresses from novice to expert through the levels of knowledge, competence, performance, and action.^4^, ^5^ This framework is illustrated in Figure 1. In all the educational opportunities described in this work, trainees first build a broad foundation of knowledge (the base of the pyramid), then advance to knowing how to complete a given procedure (a narrower level of the pyramid). They then develop to a level where they can demonstrate that they can perform the procedure in a testing environment, and finally continue to perform the task in routine practice (the apex of the pyramid). This framework emphasizes that the ultimate goal for learners in a clinical environment is for competence in the real‐world setting of practice. This pragmatic approach corresponds well with the clinical nature of medical physics service groups.

## 4 INTRODUCTION AND NARRATIVE

Northwest Medical Physics Center (NMPC) is a nonprofit organization that provides clinical physics support to over 35 radiation therapy facilities. NMPC is composed of approximately 50 physicists and dosimetrists who are based at facilities ranging from small, community‐owned cancer centers to large hospital systems offering multiple radiation treatment modalities. The primary focus of NMPC staff has been the provision of clinical physics and dosimetry support in radiation oncology, as well as project work that includes therapy and diagnostic shielding design, linear accelerator (linac) commissioning, and many types of auxiliary radiation therapy imaging and treatment systems. However, the wide range of clinical sites and associated treatment technologies also provide excellent opportunities for clinical training and research. The nonprofit status of the organization has enabled the development of a structured educational and research mission, which is complementary to its clinical services.

Three clinical training programs have been established at NMPC: a medical physics residency program accredited by the Commission on Accreditation of Medical Physics Education Programs (CAMPEP), an Applied Physics Technologist (APT) program, and a summer undergraduate internship program. In addition to these programs, a structured approach has been taken to clinical research. A partnership has been established with a major radiation oncology clinic vendor for the purposes of validating and testing new clinical devices across multiple facilities. All these programs are managed by a dedicated education and research team of four qualified medical physicists (QMPs). The expansion of the group's clinical mission to include education and research was enabled by its nonprofit status; however, these programs have enhanced clinical services, improved the quality of patient care, and are consistent with the goals of the Medical Physics 3.0 Initiative, which seeks to expand the practice of medical physics.^6^

### 4.1 Medical physics residency program

NMPC established a residency program in radiation therapy physics in 2005. This program was CAMPEP‐accredited in 2009, becoming the first nonacademic institution to achieve accreditation. The recommended guidelines for clinical medical physics residency programs are discussed in American Association of Physicists in Medicine (AAPM) Report No. 249.^7^ These recommendations provide an important basis for designing a residency program in conjunction with the standards mandated by CAMPEP.^8^ In addition to these references, AAPM's guidelines on alternative clinical training pathways for medical physicists (TG‐133^9^) were consulted for the NMPC program, especially its discussion regarding medical physics residency training. The NMPC program consists of 2 years of clinical training and shares some characteristics with a hub‐and‐spoke residency model.^10^, ^11^ Each resident completes their training across multiple clinical sites, based mostly in Alaska and Washington states. This provides exposure to many different vendor systems in radiation therapy, as well as experience of different clinic cultures and practices. Resident recruitment was performed using standard methods and relied on oral contracts, described by Antolak^12^ as “gentlemen's agreements,” since 2013. Recruitment has been performed through the MedPhys Match^13^ since 2017 when the Match became available. In the past, a single resident was enrolled in the program at a time, except for a brief period between 2014 and 2016, when the program was expanded to accommodate two residents. The program was expanded again in 2022 to accommodate two residents: one position starting in July and the other in January, with the July position selected through the MedPhys Match and application materials collected through the AAPM Medical Physics Residency Application Program (MP‐RAP). The position starting in January is recruited outside of the Match. NMPC has shown a strong preference for recruiting its own graduating residents for junior physics positions after residency. The start times for the two residency positions were staggered to better suit this recruitment demand.

**FIGURE 2:**
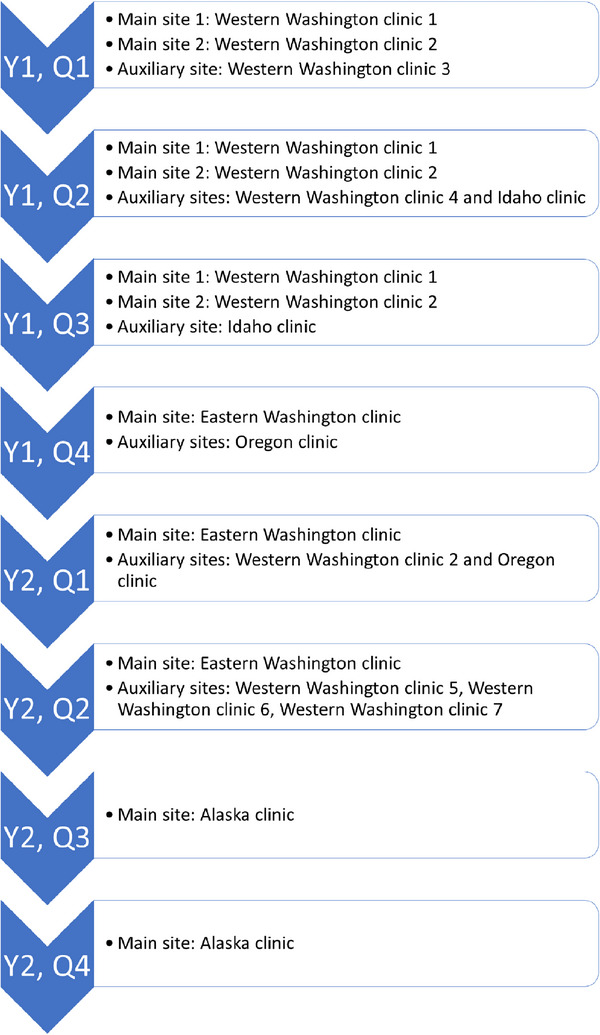
Schematic demonstrating a recent NMPC resident's schedule by year and quarter, distributed between different geographic sites. The resident was stationed at either one or two main sites for each quarter, with additional visits to auxiliary sites for shorter periods of time. These auxiliary site visits included trips for procedure observations, source exchanges, annual linac QA projects, and new linac commissioning. The shortest auxiliary site visit was 1 day, and the longest was 2 weeks. For quarters split between two main sites, the resident spent 3 days per week at one site, and 2 days per week at the other site.

**FIGURE 3:**
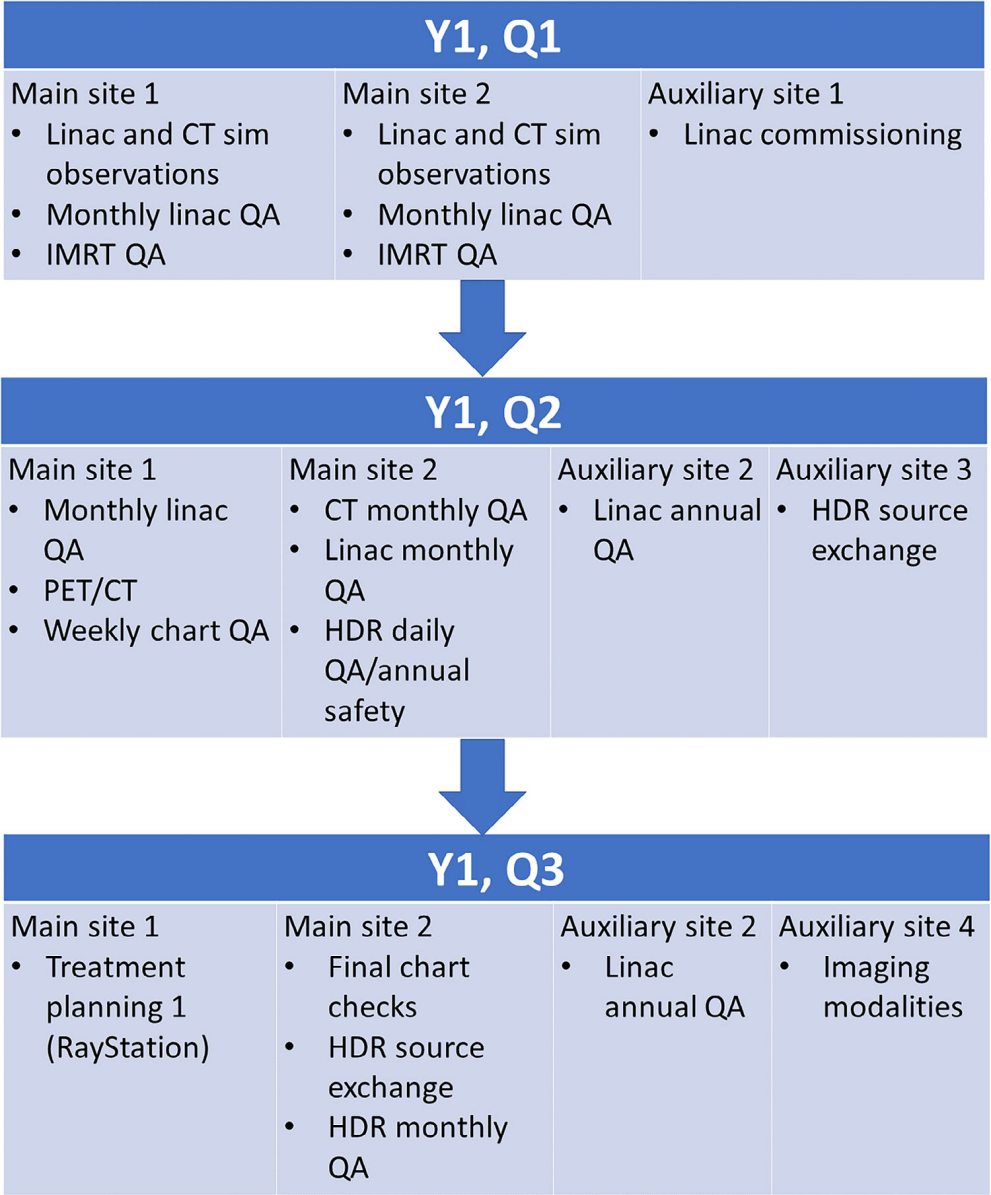
Graphic of main topics covered from the resident's schedule illustrated in Figure 2 for the first three quarters of the resident's work at NMPC. Following the third quarter, the resident proceeded to other rotations at different geographic sites, as described in Figure 2.

NMPC currently has six designated clinics for residency training, although residents visit other sites to assist with commissioning projects, system upgrades, and annual linac quality assurance (QA). Residents currently rotate through three of the designated clinics as main sites during their time in the program. The schedule of a recently graduated resident is included in Figures 2 and 3. These figures are meant to serve as an example of how a resident's geographic assignments and workload are distributed in this model of a residency. Each resident is assigned between three and five training objectives every 3 months. These objectives cover routine clinical physics work, including treatment planning, and commissioning tasks using a curriculum based on standards outlined by CAMPEP.^8^ Clinical training is performed on MOSAIQ (Elekta, Stockholm, Sweden) and ARIA (Varian Medical Systems, Palo Alto, CA) record and verify systems; treatment systems including Varian and Elekta linacs, Varian and Elekta high dose rate (HDR) brachytherapy afterloaders, and Gamma Knife (Elekta); Eclipse (Varian), Monaco (Elekta), Oncentra Brachy (Elekta) and RayStation (RaySearch Laboratories, Stockholm, Sweden) treatment planning systems; and a low‐energy dermatology system. Residents are mentored by an on‐site physicist who guides the resident through each set of objectives.

In addition to their training objectives, residents are expected to take on QA responsibilities at each clinic once they achieve an appropriate competency level. Within the first few months of starting at a clinic, residents are expected to perform weekly chart checks, monthly linac QA, patient‐specific intensity modulated radiation therapy (IMRT) QA, and daily HDR afterloader QA without personal or direct QMP supervision.^14^ By the start of their second year, they also perform initial chart checks and annual linac QA without these levels of supervision.

Per CAMPEP standards, the NMPC residency program is governed by a residency steering committee currently composed of six physics faculty and two non‐physics faculty members. Experienced physicists interested in mentoring activities typically occupy faculty positions for a 5‐ to 7‐year term. These faculty rotations allow for the provision of varied expertise and fresh perspectives to the residency program. The non‐physics faculty members, a dosimetrist and a physician, are employees of NMPC client clinics. The NMPC residency committee meets twice a year to review changes to the program and the progress of the residents.

The residents are evaluated quarterly by physics residency faculty members; each resident undergoes a 2.0−2.5 h oral exam each quarter, designed to test their clinical knowledge as well as prepare them for the American Board of Radiology certification exams. The oral exam is performed remotely using MS Teams (Microsoft, Redmond, WA), allowing the participation of faculty based at multiple, geographically distributed clinical sites. Residents are expected to submit a written report on each clinical objective prior to this exam. Any questions that the resident fails to answer satisfactorily in the exam must be addressed in a follow‐up report. In addition to these exams, each resident has bi‐weekly virtual meetings with a faculty member from a clinic other than where the resident is based for that rotation. These meetings allow faculty to determine whether the resident is meeting their training objectives and provide the resident with a regular point of contact for feedback, independent of their on‐site mentor.

The NMPC residency is self‐funded, and resident presence may be explicitly included in the legal contract language for a given clinical site. NMPC has found residents provide a welcome extra set of hands to staff physicists, many of whom express interest in the opportunity to help train residents, who will progress to become future colleagues. Clinics that may initially be somewhat skeptical about hosting residents recognize the value‐added contribution of the resident after they leave for the next rotation and look forward to hosting the next resident.

### 4.2 APT program

NMPC's APT Program was designed as a 1‐ to 2‐year training program for individuals who had recently completed an undergraduate degree in the physical sciences or engineering. This program provided the opportunity to explore a career in medical physics prior to applying to graduate school. The APT acquired hands‐on experience in radiation therapy physics, assisting QMPs in day‐to‐day responsibilities as well as larger clinical projects in busy radiation oncology departments. Prior to 2018, the NMPC APT program was an informal, ad hoc hourly paid position. The program was converted to a formal, salaried position with benefits in 2018, and a QMP was assigned as an APT manager. The intention was to improve the educational and mentorship experience of the APT program, so it would be consistent with the NMPC education mission. A single APT was enrolled in the program at a time. Recruitment efforts were targeted at 4‐year colleges with physics and engineering departments in Oregon and Washington states. The APT program was retired in 2021 due to financial constraints.

Each APT was assigned to a base clinic in Washington state, where they worked on routine clinical responsibilities, such as patient‐specific QA and linac QA, under direct or personal supervision of a QMP. The level of APT supervision was broadly based on the Medical Physics Practical Guidelines (MPPG) 3.A.^14^ Other physicists in the region were able to request the assistance of the APT, often for supervised commissioning projects in which the APT could assist with tasks such as water‐tank measurements, end‐to‐end testing, and data analysis. Like the residents, the APTs played a significant role in linac commissioning work and helped reduce the burden of routine clinical QA; however, their impact was more limited due to a smaller range of responsibilities and the larger amount of supervision required. The APT manager, typically located at a different clinical site, completed weekly check‐in calls to monitor the welfare of the APT. The manager also organized the APT clinical schedule and assisted the APT with graduate school application processes.

### 4.3 Summer internship program

NMPC has recruited undergraduate physical science and engineering students as clinical interns on an ad hoc basis since 2005. A more formal internship program was started in 2018 using the AAPM Summer Undergraduate Fellowship Program (SUFP) as a template.^15^ The NMPC summer undergraduate internship program is a 10‐week paid internship open to rising undergraduate juniors and seniors in physics, engineering, and related fields. Two interns are recruited each year, although only one intern has been recruited since 2020 due to clinic restrictions related to the COVID‐19 pandemic. It is expected that as clinics ease restrictions on nonessential visitors and trainees, the intern program will resume. Similarly to the APT program, recruitment efforts have been targeted at 2‐ and 4‐year colleges with physics and/or engineering departments in Oregon and Washington states. Each intern is based at a single clinical site for the duration of their internship, where they work on a clinical development project under the direct supervision of a QMP. Interns also receive the opportunity to observe and assist with routine QA activities such as patient‐specific IMRT QA and monthly linac QA. Examples of recent clinical projects include software coding for Winston‐Lutz image analysis, linac couch transmission measurements, optically stimulated luminescence dosimeter (OSLD) measurement and analysis, and CyberKnife measurements designed to investigate the effect of chamber orientation.^16^ An intern manager, located at another clinic, conducts weekly check‐in calls to evaluate the welfare of the intern and to provide them with another point of contact for questions and concerns. As with the residency and APT programs, the large number of physicists spread over multiple sites allows for cross‐site peer review and autonomous evaluation of the trainee. The internship provides valuable clinical exposure to undergraduate students and gives NMPC staff greater resources for completing important clinical development tasks.

### 4.4 Research and development program

Research has not been the primary focus of physics staff at NMPC, although the group has cultivated a robust performance history in clinical development going back to the institution's founding (see, e.g., Jones and Washington,^17^ Jones and Schumacher,^18^ Jones et al.,^19^ Kaurin et al.,^20^ Brown et al.,^21^ and Zaini et al.^22^). Research and development have been performed on an ad hoc basis when time and interest intersect a clinical problem. A new, structured approach to research was begun in 2018. NMPC reached out to multiple clinical vendors to inquire about forming a research and development partnership to evaluate new clinical products across different clinical sites. In 2019, the organization signed a professional consulting agreement with Varian Medical Systems to develop and test a new end‐to‐end stereotactic radiosurgery (SRS) phantom, and to recommend appropriate commissioning criteria for multileaf‐collimator (MLC) SRS treatments on Varian TrueBeam and Edge linacs.

NMPC operates as a highly decentralized group. Physics staff work in very different clinical environments, and physicists have developed distinct and innovative approaches to SRS workflow. This heterogeneous approach proved to be an excellent environment for the development of a suitable end‐to‐end testing procedure and appropriate commissioning tolerances that could be applied across many types of clinical sites. Initial testing of a prototype phantom design and development of an end‐to‐end procedure was performed at three clinical sites with well‐established SRS programs. The phantom design was evaluated based on its suitability for measuring the dosimetric and geometric features recommended for SRS end‐to‐end testing as defined by AAPM's SRS‐SBRT Medical Physics Practice Guideline.^23^ Varian revised their phantom design based on this evaluation;^24^ end‐to‐end data were then acquired with a new phantom design, across six clinical sites, to determine appropriate passing criteria for end‐to‐end tests performed on TrueBeam and Edge machines. Phantom simulation, planning, and data collection were performed by on‐site physicists, including a resident, over 9 months from 2020 to 2021. Periodic meetings, typically once a month, were held between a Varian representative and the NMPC education and research team to discuss the progress of the work.

## 5 INNOVATION

Medical physics service groups provide key services in ensuring the safe and effective delivery of treatment to radiation oncology patients. Beyond clinical support, these service groups have the ability to integrate educational and research missions into their practices. The NMPC programs described here highlight innovative ways to adapt these areas to a nontraditional, nonacademic environment. The wider impact of these efforts includes the potential to improve patient care and foster interdisciplinary collaboration.

### 5.1 Medical physics residency program

The number of yearly applicants for the NMPC residency program has varied from 44 to 142 since the program became CAMPEP accredited in 2009.^25^ Since 2009, seven physicists have completed their residency training at NMPC. Six of those physicists are now ABR‐certified in radiation therapy physics. Annually, CAMPEP‐accredited graduate programs award more medical physics master's degrees than Ph.D. degrees, but the majority of CAMPEP‐accredited medical physics residencies are more likely to match Ph.D. candidates than master's candidates during the Match selection process.^26^, ^27^ NMPC has a strong track record of recruiting excellent MS physicists: although several PhD candidates have been ranked for recruitment in the past, to date, all NMPC residents have been MS physicists. Additionally, NMPC has shown a strong preference for hiring its graduating residents; all but one former resident have been employed at NMPC as full‐time medical physicists following residency.

The NMPC residency uses the Typhon All Health Student Tracking System (Typhon Group, Metairie, Louisiana) as a cloud‐based platform for logging resident clinical activities. Typhon platforms have been used for logging competencies, assessments, feedback, and training hours for other health care providers, including physicians,^28^ physician assistants,^29^ nurse practitioners,^30^ and dental hygienists.^31^ This cloud‐based software serves as a useful tool for tracking medical physics resident training progress, as well as compiling feedback for residents and faculty and organizing records. Resident feedback on the program is sought immediately prior to steering committee meetings and via a quarterly evaluation questionnaire that is, administered through Typhon. The Radiation Oncology Education Collaborative Study Group Core Curriculum Project Leadership Committee, formally endorsed by the American Society for Radiation Oncology (ASTRO), has emphasized the need for competency‐based training for radiation oncology resident education, including the tracking of entrustable professional activities (EPAs).^32^ Similar competency‐based medical education frameworks have been used for medical physics training.^33^ Chronicling progression of entrustability, from a new resident simply observing, then progressing to performance of the activity with various levels of supervision, to finally overseeing other residents performing the activity, can be cataloged in electronic tracking systems such as Typhon as a means to verify the resident's advancement up the levels of the pyramid of clinical competency.

One of the major strengths of the NMPC residency is the incorporation of multiple clinical sites and commissioning projects to allow the resident to view not only different equipment, procedures, and workflows, but also to facilitate the resident participating in different clinic cultures (see Figures 2 and 3). External CAMPEP reviewers have indicated that the residency hosted at this institution differs from other available residency programs, specifically the wide number of sites that the residents visit and the large number of physics mentors. Residents are expected to change their living location 1‐2 times during their residency. NMPC covers relocation expenses as well as travel expenses for shorter‐term projects, including commissioning.

Because of the brisk demand for NMPC's commissioning services, residents have high involvement in linac commissioning projects. NMPC commissions approximately five to ten new linacs each year. Residents are typically involved in at least four of these projects over the course of their residency. If an estimated linac lifetime is approximately 10 years, a single program maintaining five linacs will commission a linac, on average, once every 2 years. For a resident to be involved in an average of four commissioning projects, a program would need to maintain at least twenty linacs.

Residents travel away from their base clinic for up to 2 weeks per quarter for commissioning. Residents are expected to participate in radiation surveys, linac dose calibration, beam scanning, output factor measurements, treatment planning system modeling, volumetric modulated arc therapy (VMAT) commissioning, on‐board imaging (OBI) commissioning, data‐collection automation, and end‐to‐end‐testing. These routine QA and commissioning responsibilities provide the resident with valuable clinical experience and allow them to make a significant contribution to the clinical mission of NMPC. This work helps relieve the workload of the on‐site physics team, allowing them to focus on clinical activities exclusively reserved for QMPs. It also helps demonstrate the value of the resident to clinic administration, which may be initially skeptical of hosting a trainee physicist in a nonacademic environment. The expected resident travel schedule is discussed with all residency candidates at both the initial phone interview and the final video conference interview stages, as well as at the AAPM residency fairs. NMPC's multi‐institutional approach encourages residents to experience a range of different clinical environments, and therefore shares similarities to “hub‐and‐spoke” model residencies that are designed to take advantage of multiple clinical sites. To the best of our knowledge, the majority of currently available hub‐and‐spoke model residencies are affiliated with major academic centers,^34^ and no publications detail similar residency administration through a nonacademic, nonprofit organization.

One potential challenge of a nonacademic residency program is access to publications commonly provided to residents at academic institutions, such as journal article access and books. To address this issue, NMPC pays for AAPM and ASTRO membership for its residents. These organizations offer access to peer‐reviewed journals such as *Medical Physics* and *International Journal of Radiation Oncology, Biology, Physics* as well as National Council on Radiation Protection and Measurements (NCRP) and International Commission on Radiation Units and Measurements (ICRU) publications. With the longstanding nature of the NMPC residency, a large electronic repository of required reading materials has been compiled over the years the residency has been accredited. This library of materials is limited for educational purposes within the nonprofit educational scope of the residency and is shared with incoming residents through fair‐use guidelines. Additional books, reading materials, and other resources required by the residency or for preparation for board exams are budgeted for by NMPC and reimbursed up to $500 per year per resident.

### 5.2 APT program

The number of applications for the APT program varied from 10 to 15 per year during the time period 2018−2021. Three recently graduated physics students were successfully recruited and trained during this time. APTs have presented work at national conferences^35^ as well as contributed as authors on published studies.^36^ APT alumni have since gone on to study and conduct research at accredited medical physics graduate programs, including at the University of Wisconsin, University of Victoria, Oregon Health and Science University, and Vanderbilt University. When interviewed, APT alumni all indicated they applied for the APT program with the goal of gaining clinical experience prior to applying to graduate school.

There is currently no literature available detailing the APT experience. Research in other undergraduate science, technology, engineering, and math (STEM)‐based fields has shown that immersive field experiences result in higher levels of translational outcomes and deeper levels of understanding than classroom learning or experiential learning field trips.^37^ The APT was modeled similarly to the AAPM's definition for a medical physics assistant (MPA).^38^ However, APT and MPA positions contain some key differences, specifically that the formalized APT program at its core was designed to be an educational program, with significant time spent by the educational team on mentorship and educational opportunities. The APT position required no specialized medical physics education or training, and the position was designed to be temporary prior to the candidate moving on to graduate school. In contrast, MPA positions by AAPM definition require medical physics education or training. MPA positions may also be terminal, whereas the APT position was designed to be a short‐term stepping stone prior to undergraduate physicists continuing to graduate study. As such, the APT program was designed to be both pragmatic for NMPC and beneficial for the APT. The duration of the program for each APT was mostly determined by the timing of their graduate school applications; however, all APTs have worked at NMPC for at least 1 year and no more than 2 years.

The APTs who participated in the program did not yet have extensive medical physics or general research experience at the time of admission to the program, but graduated with at least a year of real‐world clinical experience with the goal of providing a supporting foundation at the lower levels of Miller's pyramid on which to build. To do so, the APT program provided a blended educational environment including project‐based learning^39^ and mentorship regarding careers in medical physics, as well as preparation for the graduate school experience. APTs had the opportunity to develop technical skills, improve communication, and gain practical experience. This preparation included discussions regarding the “hidden curriculum” of graduate school education, as it is sometimes discussed in the literature, including expectations, unwritten rules, and implicit academic values^40^ that may not be obvious to students who may be the first in their family to attend graduate school (or to have attended college). Through exit and follow‐up interviews, APT graduates indicated that they perceived that the program gave them an advantage in competitive graduate school admissions processes and provided a solid foundational understanding of basic clinical physics for their graduate studies.

### 5.3 Summer internship program

Although recruitment for the summer undergraduate internship has been suppressed since 2020 due to COVID‐19 restrictions, the intern program has proven very popular, with as many as 45 complete applications to the program in 2019. Five undergraduate students have completed 10‐week summer internships at NMPC since 2018. Of those five students, one intern presented their project work at the 2020 Radiosurgery Society Meeting;^16^ another intern presented work at local and national AAPM meetings,^41^ was subsequently recruited as an APT, and is now enrolled in the graduate medical physics program at the University of Wisconsin; and a third intern was recruited as a MPA at NMPC following completion of their undergraduate studies.

AAPM offers similar undergraduate internship experiences through their SUFP and Diversity Recruitment through Education and Mentoring Program (DREAM), formerly the Minority Undergraduate Summer Experience Program. AAPM has offered paid support for talented undergraduates to conduct mentored research since 2001. The current SUFP and DREAM programs offer 10‐week paid internship experiences for undergraduate students by pairing each student with a mentor to guide them in medical physics research and/or clinical responsibilities. Published results showing high satisfaction rates from both the mentors and student graduates.^15^ Both the SUFP and DREAM programs attract and support exceptional undergraduate students. Research in other STEM‐based undergraduate summer internship and research programs demonstrates that students identify improvement in critical professional development areas based on these types of experiences. Student self‐identified improvement areas included laboratory techniques, time management, comfort with reading scholarly literature, and independence.^42^, ^43^, ^44^ Unfortunately, the AAPM offers a limited number of SUFP and DREAM opportunities each year, and many available mentors and institutions are not matched with student candidates. However, the success of the NMPC internship program has shown that supporting early‐career physicists through a summer undergraduate internship program is possible outside of the established AAPM program (and outside of academia), and can result in positive educational experiences for early‐career physicists.

### 5.4 Research and development program

The Varian SRS phantom project had a synergistic relationship with NMPC educational and clinical responsibilities. It allowed the intercomparison of SRS practices across multiple clinical sites. It also gave clinical sites another method for validating the integrity of their SRS treatments, which was used as a justification for spending clinic time on this project in discussions with clinic administrations. The dosimetric and geometric SRS data acquired across six linacs established baseline data for future SRS commissioning on new machines. It also served as an excellent teaching tool for one of our residents, who played a significant role in data collection for this research.

The Varian SRS phantom became commercially available for clinical users in 2021. The development and commissioning work performed with the phantom was presented at the 2021 AAPM and ESTRO annual meetings.^24^, ^45^ A paper describing the phantom design, an end‐to‐end commissioning procedure, and recommended tolerances for MLC‐based SRS treatments was published in 2022.^46^ The phantom has proved to be a valuable tool at NMPC for commissioning SRS treatments on new linacs, enhancing the organization's existing commissioning services. To date, NMPC has used the phantom for nine linac commissioning projects.

Collaborations between academia and industry across many medical and STEM‐based fields are not new, with academic‐industry partnerships (AIPs) supported with established national funding mechanisms including the National Institutes of Health's Academic‐Industrial Partnerships for Translation of Technologies for Diagnosis and Treatment R01 Research Project Grant.^47^ More specifically, these types of relationships have been leveraged between industry and medical physics academic institutions.^48^ AIPs have also been shown to provide educational benefits to undergraduate students, with successful partnerships between medical device manufacturers and biomedical engineering undergraduate students.^49^ Outside major academic centers, less common are medical physics clinical service group partnerships with industry. However, as the above discussion illustrates, these types of collaborations can be beneficial for both the industry partner as well as the physics group, and they can provide valuable educational experiences for trainees. Based on the success of the SRS phantom project, NMPC intends to continue these types of collaborations in the future.

## 6 CONCLUSIONS

NMPC has successfully established three formal clinical training programs. A structured research and development program has also been established in collaboration with a major clinical vendor. Notwithstanding the retirement of the APT program and the COVID‐19 restrictions placed on the internship program, all these programs have made a significant contribution to the clinical service mission of NMPC, as well as providing training opportunities for early‐career medical physicists across many different clinical environments. These programs are described using the widely recognized lens of Miller's Pyramid, in which learners (in this case, early‐career physicists) build from a foundation of acquiring new knowledge and skills, proceed to demonstrating understanding of that knowledge, then progress to demonstrating how to apply concepts in practice, and finally, performing independently in real‐world situations. Clinical medical physicists can and should seek to expand their traditional role, especially in the context of a shortage of QMPs^50^ and a work environment that is changing rapidly due to the introduction of artificial intelligence tools.^51^ Education and research are obvious routes for expansion. This paper has demonstrated that these subjects, often defined by traditional academic institutions, can be incorporated into nonacademic clinical environments, improving the quality of patient care, and increasing the number and type of training opportunities available for medical physicists.

## AUTHOR CONTRIBUTIONS

All the authors have made significant contributions to the design and administration of the education and research programs described in this paper. Jessica Fagerstrom and Thomas Brown were responsible for writing the paper. All authors have reviewed the paper and were given the opportunity to provide edits prior to submission.

## CONFLICT OF INTEREST STATEMENT

The authors declare no conflicts of interest.
